# Performance of Fujifilm Dengue NS1 Antigen Rapid Diagnosis Kit Compared to Quantitative Real-Time Polymerase Chain Reaction

**DOI:** 10.3390/pathogens13090818

**Published:** 2024-09-23

**Authors:** Mya Myat Ngwe Tun, Merveille Kapandji, Atsuhiko Wada, Ko Yamamoto, Shyam Prakash Dumre, Khine Mya Nwe, Htin Lin, Yuki Takamatsu, Kyaw Zin Thant, Hlaing Myat Thu, Takeshi Urano, Basu Dev Pandey, Kouichi Morita

**Affiliations:** 1 Department of Tropical Viral Vaccine Development, Institute of Tropical Medicine, Nagasaki University, Nagasaki 852-8523, Japan; 2 Department of Virology, Institute of Tropical Medicine, Nagasaki University, Nagasaki 852-8523, Japan; mckkapandji@gmail.com (M.K.); drkhinemyanwe@gmail.com (K.M.N.); yukiti@nagasaki-u.ac.jp (Y.T.); 3 Center for Vaccines and Therapeutic Antibodies for Emerging Infectious Diseases, Shimane University, Izumo 690-8504, Japan; turano@med.shimane-u.ac.jp; 4 Medical Systems Research and Development Center, FUJIFILM Corporation, Tokyo 107-0052, Japan; atsuhiko.wada@fujifilm.com (A.W.); ko.yamamoto@fujifilm.com (K.Y.); 5 Central Department of Microbiology, Tribhuvan University, Kathmandu 44601, Nepal; sp.dumre@gmail.com; 6 Department of Medical Research, Ministry of Health, Yangon 11191, Myanmar; drhtinlin@gmail.com (H.L.); hmyatthu28@gmail.com (H.M.T.); 7 Myanmar Academy of Medical Science, Yangon 11201, Myanmar; drkz.thant@gmail.com; 8 DEJIMA Infectious Diseases Research Alliance, Nagasaki University, Nagasaki 852-8523, Japan; drbasupandey@gmail.com

**Keywords:** dengue virus, Fujifilm NS1 kit, SD Bioline NS1 kit, qRT-PCR

## Abstract

Dengue is a viral infection caused by the dengue virus (DENV), transmitted to humans through the bite of infected *Aedes* mosquitoes. About half of the world’s population is now at risk of dengue, which represents a global public health concern, especially in tropical and subtropical countries. Early detection of the viral infection is crucial to manage the disease; hence, effective rapid diagnostic tests are essential. In this study, we evaluated the performance between the new Fujifilm Dengue non-structural antigen diagnosis kit (FF NS1 kit) and the SD Bioline NS1 antigen test kit (SD NS1 kit) against the quantitative real-time polymerase chain reaction (qRT-PCR) assays. The 140 acute serum samples collected from the Yangon General Hospital and Yangon Children’s Hospital, Myanmar, from 2017 to 2019 were characterised by the three assays. With the qRT-PCR as the standard, the FF NS1 kit and the SD NS1 kit exhibited sensitivity of 94.3% and 88.6%, respectively, and specificity of 100% in both kits. Moreover, the positivity rates of the FF NS1 kit and the SD NS1 kit were 97.5% and 95% in primary infection and 90% and 80% in secondary infection, respectively. Our overall results suggest that the FF NS1 kit is reliable and accurate for detecting DENV infection.

## 1. Introduction

Dengue is caused by the dengue virus (DENV) and is considered the most important and prevalent arboviral infection in the world. Approximately 390 million dengue infections occur annually worldwide, with 96 million exhibiting clinical manifestations [1,2]. This mosquito-borne high-burden disease is endemic in tropical and subtropical regions of the world [3]. The DENV belongs to the *Flavivirus* genus, which includes many other human pathogens of clinical importance, such as yellow fever virus, Zika virus (ZIKV), and West Nile and Japanese encephalitis viruses (JEV). DENV is transmitted through the bite of an infected *Aedes* mosquito during a blood meal and its genome consists of a single-stranded positive-sense RNA encoding for three structural proteins (capsid, prM/M, and E) and seven non-structural proteins (NS1, NS2A, NS2B, NS3, NS4A, NS4B, and NS5) [4,5]. DENV has four antigenically distinct serotypes (DENV1-4) that share up to 65% of the viral genome. An infection with each serotype provides lifelong immunity for the causative serotype but not for the others. Furthermore, secondary infections caused by one of the four serotypes are usually more severe than primary infections [6,7].

Clinical manifestations of DENV infections range from a mild febrile-like illness to a classic dengue fever or the most severe form of the disease (dengue haemorrhagic fever (DHF)) and dengue shock syndrome (DSS)). Population-based studies have suggested that the main outcome of DENV exposure is asymptomatic infection. Nevertheless, DHF is associated with high morbidity and mortality [8]. During the acute phase of dengue infection, the fastest way to confirm infection by laboratory diagnosis is through detection of the virus genome by molecular tests such as quantitative reverse transcription-polymerase chain reaction (qRT-PCR) or the conventional RT-PCR. However, most developing countries lack the facilities required for diagnosing dengue using molecular means; instead, they use the serological methods. Among serological techniques, the detection of the dengue NS1 antigen and anti-dengue IgM and IgG antibodies is commonly used and several assays are available, ranging from capture enzyme-linked immunosorbent assays (ELISAs) to rapid diagnostic tests (RDTs) based on immune chromatographic or immunoblot technologies. Unlike the molecular tests, most serologic tests detect dengue infection at a later stage of the disease. The serological tests that can detect early infection involve the detection of the NS1 antigen. Detection of this antigen allows dengue diagnosis for a timely implementation of supportive therapy and monitoring, consequently reducing and limiting the risk of complications such as DHF or DSS, particularly in dengue-endemic countries [9].

Dengue is a public health concern in Myanmar. The country experiences seasonal outbreaks of dengue fever, with a higher incidence during the rainy season when mosquito populations tend to increase. Although dengue cases have been reported throughout the country, urban areas and cities are particularly affected [10]. A rapid, sensitive, and specific diagnosis of DENV infection is needed for appropriate patient management at the early phase of infection. Assays involving the detection of NS1 viral antigen are useful for early diagnosis of dengue because high concentrations of the NS1 protein are present in the blood of patients during the acute phase of both primary and secondary infections and the protein is detectable for up to 9 days after the onset of symptoms [3]. DENV NS1 RDT kits are manufactured for easy and fast diagnosis and are useful for point-of-care tests, particularly in areas with limited diagnostic capabilities. Depending on the brand, it takes 10–25 min to perform the test, which is easy to use and interpret. The kits can be kept at room temperature. Several brands of rapid tests are available for purchase in developing countries where dengue is endemic. In the present study, we assessed a newly developed rapid test kit, the Fuji NS1 RD kit, for its accuracy, sensitivity, and specificity to ensure that it meets the standards for laboratory diagnosis. This new kit was compared with SD Bioline NS1 RDT kit, which is widely used in dengue endemic countries. It was also compared with qRT-PCR as the current standard, which provides information on the infecting serotype and virus copies of DENV of acute serum samples. We used serum samples from dengue infected patients in a DENV-endemic region in Myanmar when evaluating the new kit.

## 2. Materials and Methods

### 2.1. Study Population

One hundred and forty serum samples collected from children and adults treated in two hospitals, Yangon General Hospital and Yangon Children’s Hospital, in Yangon, Myanmar from June 2017 to December 2019 were used to analyse the newly developed kit. Following the 2009 WHO guidelines, patients were classified as dengue without warning signs (DWoWS), dengue with warning signs (DWWS), or severe dengue (SD) on the basis of clinical findings and laboratory tests. All patients visited the hospital 1 to 7 days after onset of fever, and their serum samples were collected on their first visit or admission day.

### 2.2. Rapid Diagnostic Tests

Serum samples kept at at −80 °C ultra-low freezer were thawed for use for the three types of tests, which were conducted successively or simultaneously on the same day. Initially, the samples were examined by Bioline DENV Duo kit (Abbott Diagnostics Inc., Seoul, Korea). Subsequently, other volumes of the same samples were analysed by using the Fujifilm Dengue NS1 antigen rapid test kit. Other volumes of the same samples were subjected to qRT-PCR. Serological results were then compared with dengue specific qRT-PCR results to assess the sensitivity and specificity of the Fujifilm Dengue NS1 antigen rapid test kit. Details of the procedures for SD Bioline Dengue NS1 kit, Fujifilm Dengue NS1 kit, and qRT-PCR are described below.

### 2.3. SD Bioline Dengue NS1 Kit

The SD Bioline Dengue Duo rapid test kit is produced by Abbott Diagnostics and is a one-step immunochromatographic assay designed for the detection of both the DENV NS1 antigen and differential DENV IgM/IgG antibodies in clinical samples such as human whole blood, serum, or plasma [11]. The SD bioline Dengue Duo rapid test contains two test devices that are positioned next to each other; the device on the left side is for the dengue NS1 antigen test, whereas the one on the right side is for the dengue IgG/IgM test. In this study, we used the SD Bioline to identify positive DENV infection by NS1 antigen alone. We describe detection of the dengue NS1 antigen as follows: the clinical sample is captured by the anti-dengue NS1 Ag-colloid gold conjugate in the device and this complex migrates along the length of the device; it is then captured by the anti-dengue NS1 antigen immobilized on the membrane strips. This results in the generation of a colour line to show positive detection. All tests in this study were carried out in accordance with the manufacturer’s instructions. Briefly, 100 μL of the test sample was added into the sample well and test result was interpreted after 15–20 min.

### 2.4. Fujifilm Dengue NS1 Kit

**Figure 1:**
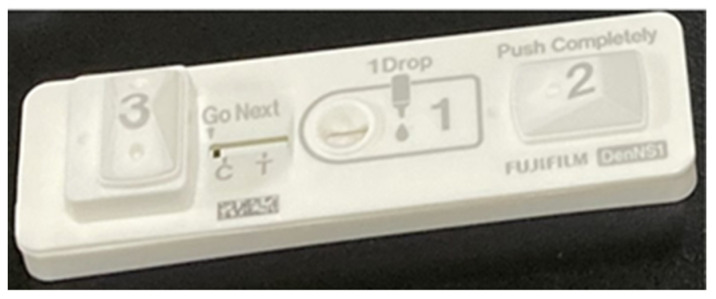
Schematic illustration of the Fujifilm Dengue NS1 rapid antigen test.

Fujifilm Dengue NS1 antigen rapid test kit detects the DENV NS1 antigen in human whole blood, serum, or plasma to confirm a DENV infection. To describe the procedure briefly, anti-dengue NS1 monoclonal antibody and anti-mouse IgG were diluted in 50 mM Tris buffer (pH 7.0) and dropped onto a nitrocellulose membrane to obtain the test and control line, respectively. The membrane was then dried at 50 °C for 30 min to immobilise antibodies. A reagent pad containing anti-dengue NS1 monoclonal antibody conjugated with colloidal gold was placed in the middle of the test strip (upstream from the test line). A test strip, a water adsorption pad, and two sealed pots containing 185 µL of Reagent A (an aqueous reducing-agent solution containing 0.47 mol/L Fe (NH_4_)_2_(SO_4_)_2_ and 0.15 mol/L HNO_3_) and 185 µL of Reagent B (0.31 mol/L AgNO_3_ and 0.06 mol/L HNO_3_) were placed in a cartridge. The schematic illustration of Fujifilm dengue NS1 kit is shown in Figure 1. A volume of 24 μL of the test sample was required for NS1 detection and the test result was interpreted after 15–20 min.

### 2.5. Determination of Primary and Secondary Infections

The infection status of donor patients was determined by in-house anti-DENV IgG antibody indirect ELISAs whereby the optical density (OD) values were read at 492 nm [12]. If the IgG titre was ≥29,000 or <29,000, infection was considered secondary or primary, respectively, following the cutoff values we used in our laboratory. These values highly correlated with the WHO gold standard, the dengue hemagglutination inhibition test, as shown in the previous study in our laboratory [12].

### 2.6. Viral RNA Extraction and qRT-PCR

To confirm the presence of DENV and other flaviviruses such as JEV and ZIKV in the serum, viral RNA was extracted using a QIAmp Viral RNA Mini kit (cat. no. 52906, Qiagen, Hilden, Germany), following the manufacturer’s instruction. Then qRT-PCR was performed on the RNA, using TaqMan Fast Virus 1-Step Master Mix (cat. no. 4444436, Life Technologies, Carlsbad, CA, USA) along with the DENV, JEV, and ZIKV primers and probes (Supplementary Table S1), which were reported in previous studies [13,14,15]. The reaction mixture consisted of 4 μL of water, 2.5 μL of TaqMan Fast Virus 1-Step Master Mix (2×), 0.5 μL of each primer (10 mM), 0.25 μL of the probe with envelope gene of dengue serotype specific primers, and 5 μL of RNA (10–50 ng/μL). Amplification was performed for 5 min at 50 °C, followed by 20 s at 95 °C and, finally, 40 cycles at 95 °C for 3 s and 60 °C for 30 s. Ten-fold serial dilutions of standard RNA (10^2^–10^8^ genome copies) were applied for the quantification of viral genome levels. The detection limit for the viral genome was 100 copies, and the viral genome levels were expressed as log10 genome copies/mL.

### 2.7. Statistical Analysis

Microsoft Excel was used to enter data. Data analysis was performed by using the software—MedCalc—version 22.039 (Ostend, Belgium). Sensitivity, specificity, positive predictive value (PPV), negative predictive value (NPV), positive likelihood ratio (PLR), and negative likelihood ratio (NLR) with 95% confidence intervals (CIs) were analysed. The McNemar test was used to compare sensitivities of two diagnostic assays.

## 3. Results

### 3.1. Characteristics and Distribution of DENV Serotypes

By using qRT-PCR results as the reference diagnostics for DENV infection, 140 serum samples were analysed. Out of 140 serum samples, 70 samples were from clinically dengue suspected patients, and they were determined to be dengue positive by qRT-PCR. The remaining 70 samples were from the patients who showed no clinical symptoms of dengue and they were DENV, ZIKV, JEV negative by qRT-PCR. Among dengue-positive samples, 40/70 were from patients with primary infections and 30/70 were from those with secondary infections. All four dengue serotypes were detected, as follows: DENV-1, 25 samples (35.7%); DENV-2, 8 samples (11.4%); DENV-3, 15 samples (21.4%); DENV-4, 22 samples (31.4%).

### 3.2. Performance of the Dengue Rapid Diagnostics Test Kits Compared to the Reference Test (qRT-PCR)

Based on the reference qRT-PCR results, the clinical performance of the Fujifilm Dengue NS1 antigen rapid test kit (FF NS1 Kit) was evaluated and compared with the SD Bioline Dengue NS1 kit (SD NS1 Kit) (Table 1). The demographic profile (age, sex), clincal severity, days of fever, infecting dengue serotype, viral RNA copies/reaction, type of infection, and the results of FF NS1 kit and SD NS1 kit of 70 dengue-positive patients are described in Table 2. Out of 70 qRT-PCR dengue-positive samples, 66 were positive in the FF NS1 kit and 62 were positive in the SD NS1 kit. Thus, of the two kits, the FF NS1 kit showed sensitivity of 94.3%, whereas the SD NS1 kit showed 88.6% sensitivity compared to the reference qRT-PCR results; however, no statistically significant difference were found between both kits (*p* = 0.125). The demographic profile and the results of FF NS1 kit and SD NS1 kit of 70 dengue negative patients are described in Supplementary Table S2. All of the 70 qRT-PCR dengue-negative samples were also negative in the FF NS1 and SD NS1 kits. The FF NS1 and SD NS1 antigen kits exhibited 100% specificity and had outstanding PPV and NPV values, suggesting that both kits are reliable for DENV infection diagnosis (Table 1).

### 3.3. Analysis of Test Sensitivity Based on Days of Fever

**Figure 2:**
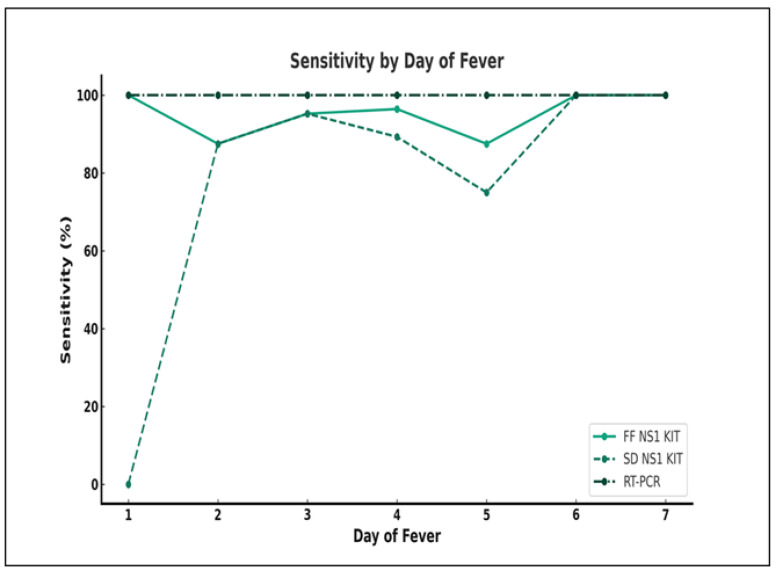
Comparison of the sensitivity of the three tests (Fujifilm NS1 kit, SD Bioline NS1 kit, and qRT-PCR) according to days of fever.

The sensitivitities of FF NS1 and SD test kits were analysed per day of fever and compared with the reference qRT-PCR results (Figure 2). The number (%) of NS1 positive sample/number of samples tested by FF NS1 from Day 1 to Day 7 of fever is as follows: 1/1 (100%), 7/8 (88%), 20/21 (95%), 27/28 (96%), 7/8 (88%), 2/2 (100%), and 2/2 (100%); those tested by SD test kit is as follows: 0/1 (0%), 7/8 (88%), 20/21 (95%), 25/28 (89%), 6/8 (75%), 2/2 (100%), and 2/2 (100%). The FF NS1 kit showed high sensitivity (>90%) that remained stable across the days of fever; the sensitivity of this kit was close to that of the qRT-PCR. The sensitivity of the SD NS1 kit varied across the days of fever compared to the FF NS1 kit and the qRT-PCR.

### 3.4. Evaluation of the Tests’ Diagnostic Ability Compared to the Reference qRT-PCR Results

**Figure 3:**
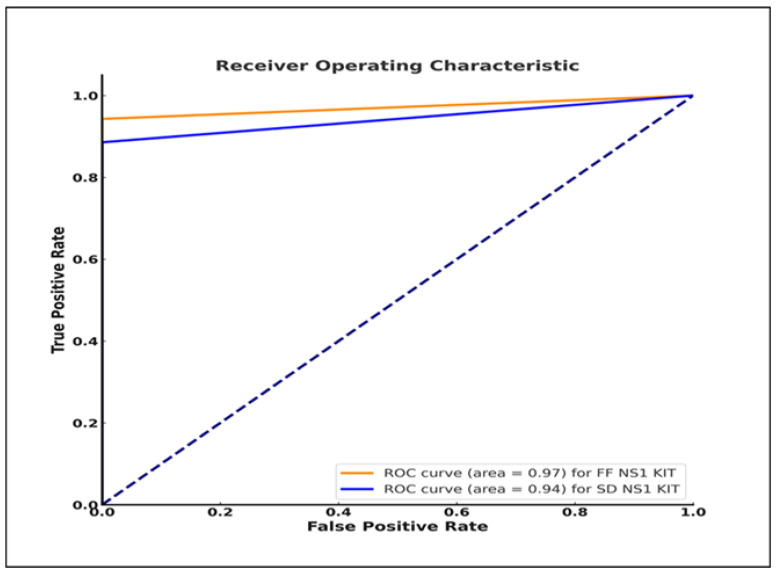
Receiver-operating characteristics (ROC) curves of the Fujifilm NS1 and SD Bioline NS1 kits. ROC dotted line from bottom left (0, 0) to upper right (1, 1) represents a strictly random classifier.

We assessed the ability of each test to diagnose and differentiate between dengue-positive and dengue-negative patients using the receiver-operating characteristic (ROC) curve, which provides a graphical representation of the diagnostic ability of each test, and the area under the curve (AUC), which shows the overall performance of each test. An AUC value of 1 indicates a perfect diagnostic ability, whereas an AUC value of 0.5 indicates a lack of diagnostic ability [16,17]. The FF NS1 kit demonstrated an AUC value of 0.97 (Figure 3) (close to 1, indicating excellent performance) while the SD NS1 kit had an AUC value of 0.94. Thus, both kits can distinguish between people with DENV infection and those without.

### 3.5. Positive Rates of the Two NS1 Kits with Respect to the Type of Dengue Infection

Among the 40-primary infection qRT-PCR-positive dengue cases, the FF NS1 kit identified 39 (97.5%) cases and the SD NS1 identified 38 (95%) cases (Table 3). Among the 30-secondary infection qRT-PCR-positive dengue cases, the FF NS1 kit identified 27 (90%) cases, whereas the SD NS1 kit identified 24 (80%) cases (Table 3). Thus, the two NS1 kits showed higher sensitivity for primary infection than for secondary infection. The sensitivities between the FF NS1 kit and the SD NS1 kit for primary and secondary infections were not significant different. (*p* = 0.307 for FF NS1 vs. Primary/Secondary infection; *p* = 0.07 for SD NS1 vs. Primary/Secondary infection).

### 3.6. Comparison between the Two NS1 Kits and Viremia Levels

As shown in Supplementary Figure S1, viremia levels present in the serum samples of patients were plotted against the infecting serotype and the type of infection of the patients with respect to each infecting serotype. Results of NS1 detection in the serum samples by using FF NS1 and SD NS1 kits are also shown in the figure. Most samples tested positive by the two kits occupied a wide range of viremia levels. The lowest viremia level for the four DENV serotypes with positve results in the two NS1 kits was approximately 10^4^ viral RNA copies/reaction (10^6^ copies/mL); this demonstrates the ability of the two kits to detect the presence of DENV through its NS1 protein in patient serum. For those infected with DENV-1, DENV-2, DENV-3, and DENV-4 serotypes, the lowest viral loads the two NS1 kits could provide positive results for in their samples were 1.2 × 10^4^ copies/reaction, 1.4 × 10^4^ copies/reaction, 1.0 × 10^4^ copies/reaction, and 1.2 × 10^4^ copies/reaction (Table 2). A correlation between viral RNA copy level in serum of patients and the infecting DENV serotype demonstrated that the viral RNA copies in DENV-1 and DENV-4 were significantly higher than DENV-2 and DENV-3 (Supplementary Figure S1A). However, the viral RNA copy levels between primary and secondary infection due to the same infecting DENV serotype were not significantly different (Supplementary Figure S1B). These results indicate that the DENV detection limit is consistent for all DENV serotypes for both kits.

## 4. Discussion

In this study, we evaluated the usefulness and applicability of the dengue FF NS1 RDT compared with the SD NS1 RDT and qRT-PCR as the standard detection method. The volume of serum required was 24 μL and 100 μL for the FF NS1 kit and the SD NS1 kit, respectively, and this showed an advantage for the FF NS1 kit over the SD NS1 kit. A single serum specimen was used during the acute phase of DENV infection. The efficient and accurate diagnosis of dengue is essential for clinical care; thus, evaluating NS1 RDTs is crucial in dengue-endemic countries. To determine the sensitivity of the RDT of interest, we selected dengue qRT-PCR-positive samples. The FF NS1 kit was evaluated in samples with DENV from the four different serotypes. To assess the specificity of the RDT, we tested samples from non-dengue febrile patients who tested negative for dengue by qRT-PCR. Those non-dengue febrile patients were negative by qRT-PCR for ZIKV and JEV, which have been circulating in Myanmar.

The diagnostic accuracy of NS1 RDT has been reported in numerous studies. In agreement with the literature, the FF NS1 kit exhibited high sensitivity (94.3%) compared to the reference qRT-PCR assay [18,19,20,21,22]. The SD NS1 kit showed 88.6% sensitivity, which agrees with the sensitivity values 73–90% reported by previous studies [22,23,24]. Out of 70 dengue qRT-PCR positive patients, 4 patients positive by FF NS1 kit were negative by SD NS1 kit. Two of the four patients were males with ages 5.3 and 11.0 years, the other two patients were females with ages 6.5 and 13.0 years. No age or gender specific differences for DENV detection by these two kits were observed. The FF NS1 and SD NS1 kits exhibited 100% specificity, suggesting that both tests identified all individuals who did not have DENV infection (i.e., no false positives).

In most studies, the NS1 antigen was detected on Days 1–9 after the onset of fever [24,25]. Here, in agreement with previous reports, both RDT kits detected the NS1 antigen in the sera of patients collected on Days 1–7 after the onset of fever. The sensitivity of the SD NS1 kit varied on different days of fever. This variation suggests that the effectiveness of the SD NS1 test could be affected by the illness stage. Furthermore, the IgG and IgM sensitivities [25,26] indicative of the immune response increased over time, reflecting their role in detecting the later stages of DENV infection. This highlights the importance of choosing the appropriate diagnostic tool for accurately detecting DENV infection at different stages of the disease.

In this study, both NS1 kits were more sensitive in detecting primary infection than secondary infection, which agrees with other reports from dengue-endemic countries [18,19,27,28,29]. In secondary dengue infection, IgG and IgM develop faster and much more highly than in primary infection. Higher IgG and IgM levels found during the acute serum in secondary infection may result in NS1 antigen-antibody immune complexes. Thus, NS1 antibody in the sample could mask NS1 binding in the RDT, leading to reduced sensitivity in secondary DENV infection [30,31,32].

Both kits showed similar sensitivities regarding the lowest viremia detection level across the different DENV serotypes, with the lowest virus detection levels fluctuating slightly. This suggests that both tests can detect the virus at various concentrations. Furthermore, no significant difference was observed in the sensitivity of both tests, which seemed to perform similarly well in detecting DENV at any given concentration. Both tests detected the virus at a concentration as low as 1 × 10^6^ copies/mL, indicating similar sensitivity at this viral load. These results correlate with other studies that reported that the detection limit for DENV in NS1 test kits varies between 10^6^–10^9^ copies/mL [21].

In conclusion, the FF NS1 kit is a reliable tool for detecting DENV infection. This finding could lead to better patient outcomes and more efficient healthcare resource allocation. The initial evaluation of the FF NS1 kit in the 140 serum samples showed promise for the usefulness of this point of care kit; however, there is still a need to confirm our results with larger sample numbers in the future.

## Figures and Tables

**Table 1:** Comparison of the performances of two dengue virus (DENV) rapid diagnosis tests. The FujiFilm NS1 kit (FF NS1 kit) and the Standard Bioline NS1 kit (SD NS1 kit) were compared by using the reference quantitative reverse-transcriptase polymerase chain reaction (qRT–PCR) as the gold standard.

Test Kit	Sensitivity% (95% CI)	Specificity% (95% CI)	PPV% (95% CI)	NPV% (95% CI)	PLR (95% CI)	NLR (95% CI)	Test Accuracy (95% CI) *	Test Concordance (k)	*p*-Value (Comparing Sensitivities)
**FF NS1 Kit**	94.3% (86.2–97.8)	100% (94.8–100)	100% (94.5–100)	94.6% (86.9–97.9)	∞	0.057 (0.02–0.14)	97.1 (92.9–99.2)	0.94, *p* < 0.001	0.125
**SD NS1 Kit**	88.6% (79.0–94.1)	100% (94.8–100)	100% (94.2–100)	89.8% (81.0–94.7)	∞	0.114 (0.06–0.21)	94.3 (89.1–97.5)	0.89, *p* < 0.001	

PPV: Positive Predictive value, NPV: Negative Predictive value, PLR: Positive Likelihood Ratio, NLR: Negative Likelihood Ratio. * Assumed 50% disease prevalence. k = Kappa agreement score indicates how similar are the two tests compared to gold standard qRT-PCR.

**Table 2:** The demographic, clinical, and viral characteristics of dengue-positive patients.

Sample ID	Sex	Age (Years)	Diagnosis	Dengue Serotype	Days of Fever	Viral RNA Copies/Reaction	FF NS1 Kit	SD NS1 Kit	Primary (P) or Secondary (S) Infection
1	M	7.0	DWoWS	DENV-1	3	1.8 × 10^5^	+	+	P
2	F	8.0	DWWS	DENV-1	3	7.1 × 10^5^	+	+	P
3	F	11.0	SD	DENV-1	4	3.5 × 10^4^	+	+	P
4	M	2.0	DWWS	DENV-1	5	4.2 × 10^4^	+	+	P
5	F	7.3	SD	DENV-1	4	1.1 × 10^5^	+	+	S
6	M	14.0	DWWS	DENV-1	4	6.2 × 10^4^	+	+	P
7	F	11.1	DWWS	DENV-1	4	5.6 × 10^4^	+	+	S
8	F	15.0	DWoWS	DENV-1	5	7.7 × 10^5^	+	+	S
9	F	13.0	DWoWS	DENV-1	2	5.2 × 10^4^	+	+	P
10	M	9.0	DWoWS	DENV-1	4	1.2 × 10^4^	+	+	P
11	F	9.0	DWoWS	DENV-1	4	3.8 × 10^6^	+	+	P
12	F	2.5	DWoWS	DENV-1	3	3.5 × 10^5^	+	+	P
13	F	9.0	DWoWS	DENV-1	4	2.3 × 10^7^	+	+	P
14	M	30.0	DWoWS	DENV-1	4	3.7 × 10^4^	+	+	P
15	M	18.0	DWoWS	DENV-1	2	8.8 × 10^5^	+	+	P
16	M	20.0	DWoWS	DENV-1	3	1.1 × 10^6^	+	+	P
17	M	22.0	DWoWS	DENV-1	2	2.3 × 10^7^	+	+	P
18	M	9.0	DWWS	DENV-1	4	5.7 × 10^6^	+	+	P
19	F	12.0	SD	DENV-1	3	6.0 × 10^6^	+	+	P
20	F	7.5	DWoWS	DENV-1	5	1.9 × 10^4^	+	+	P
21	F	9.0	DWWS	DENV-1	4	1.2 × 10^7^	+	+	P
22	M	11.0	DWWS	DENV-1	4	2.2 × 10^4^	+	+	P
23	M	5.0	DWoWS	DENV-1	4	1.6 × 10^4^	−	−	P
24	F	9.0	DWWS	DENV-1	4	1.0 × 10^6^	+	+	S
25	M	11.0	DWWS	DENV-1	2	8.1 × 10^4^	−	−	S
26	F	12.0	DWWS	DENV-2	3	1.4 × 10^4^	+	+	S
27	M	3.0	DWWS	DENV-2	5	1.3 × 10^4^	−	−	S
28	M	16.0	DWWS	DENV-2	4	2.1 × 10^4^	+	+	S
29	M	8.0	DWWS	DENV-2	3	1.6 × 10^4^	−	−	S
30	M	5.3	DWoWS	DENV-2	4	2.6 × 10^4^	+	−	S
31	M	11.0	DWoWS	DENV-2	1	4.4 × 10^5^	+	−	P
32	M	3.0	DWWS	DENV-2	4	9.7 × 10^4^	+	+	P
33	F	6.0	DWWS	DENV-2	3	2.5 × 10^4^	+	+	P
34	M	4.0	DWoWS	DENV-3	3	2.2 × 10^4^	+	+	P
35	F	8.0	DWWS	DENV-3	3	2.2 × 10^5^	+	+	P
36	M	16.0	DWoWS	DENV-3	3	5.6 × 10^4^	+	+	P
37	M	7.0	DWoWS	DENV-3	4	5.0 × 10^5^	+	+	P
38	M	8.0	DWWS	DENV-3	3	1.3 × 10^5^	+	+	S
39	M	11.0	DWoWS	DENV-3	4	3.4 × 10^4^	+	+	P
40	M	10.0	DWWS	DENV-3	3	2.7 × 10^4^	+	+	P
41	F	15.0	DWWS	DENV-3	4	1.5 × 10^5^	+	+	P
42	M	15.0	DWWS	DENV-3	4	1.0 × 10^4^	+	+	S
43	M	23.0	DWoWS	DENV-3	2	6.6 × 10^4^	+	+	P
44	M	15.0	DWoWS	DENV-3	6	2.8 × 10^4^	+	+	P
45	M	17.0	DWWS	DENV-3	3	2.2 × 10^4^	+	+	P
46	M	8.0	DWWS	DENV-3	3	4.3 × 10^5^	+	+	P
47	M	11.0	DWWS	DENV-3	7	5.2 × 10^4^	+	+	P
48	M	5.5	DWoWS	DENV-3	5	5.9 × 10^4^	+	+	P
49	F	8.0	SD	DENV-4	4	3.7 × 10^6^	+	+	S
50	M	15.0	DWoWS	DENV-4	4	3.0 × 10^5^	+	+	S
51	M	17.0	DWoWS	DENV-4	2	1.5 × 10^5^	+	+	S
52	F	9.0	DWWS	DENV-4	7	1.2 × 10^4^	+	+	S
53	F	6.5	SD	DENV-4	4	7.1 × 10^4^	+	−	S
54	M	10.0	DWWS	DENV-4	5	3.6 × 10^5^	+	+	S
55	F	27.0	DWoWS	DENV-4	2	1.9 × 10^6^	+	+	S
56	M	14.0	DWWS	DENV-4	4	2.9 × 10^5^	+	+	S
57	M	14.0	DWoWS	DENV-4	3	9.0 × 10^5^	+	+	S
58	M	13.0	DWWS	DENV-4	4	2.2 × 10^6^	+	+	S
59	M	9.0	DWoWS	DENV-4	6	1.6 × 10^5^	+	+	S
60	F	6.7	DWoWS	DENV-4	3	6.3 × 10^5^	+	+	S
61	F	2.8	DWoWS	DENV-4	3	4.0 × 10^6^	+	+	S
62	F	23.0	DWoWS	DENV-4	2	1.5 × 10^7^	+	+	S
63	F	14.0	DWWS	DENV-4	4	5.7 × 10^6^	+	+	S
64	M	7.0	SD	DENV-4	3	3.0 × 10^7^	+	+	P
65	M	8.0	DWWS	DENV-4	4	3.0 × 10^6^	+	+	P
66	F	12.0	DWoWS	DENV-4	3	2.1 × 10^6^	+	+	P
67	M	25.0	DWoWS	DENV-4	4	1.2 × 10^4^	+	+	S
68	F	22.0	DWoWS	DENV-4	5	9.3 × 10^4^	+	+	S
69	M	11.0	DWoWS	DENV-4	3	1.2 × 10^4^	+	+	P
70	F	13.0	SD	DENV-4	5	1.8 × 10^4^	+	−	S

DWoWS = dengue without warning signs, DWWS = dengue with warning signs, SD = severe dengue, M = male, F = female, FF NS1 kit = Fujifilm NS1 kit, SD NS1 kit = Standard Bioline NS1 kit.

**Table 3:** Number of patients with primary or secondary infections found dengue positive by FF NS1 kit or SD NS1 kit.

Type of Infection	Number of Patients	Number of Patients with Infection Status (%, 95% CI)	
		FF NS1 Kit	SD NS1 Kit
**Primary**	40	39 (97.5%, 92.7–100%)	38 (95%, 88.2–100%)
**Secondary**	30	27 (90%, 79.3–100%)	24 (80%, 65.7–94.3%)

FF NS1 kit = Fujifilm NS1 kit. SD NS1 kit = Standard Bioline NS1 kit. Primary = primary dengue infection. Secondary = secondary dengue infection.

## Data Availability

The datasets generated and/or analyzed during the current study are available in the manuscript and the Supplementary Materials.

## Supplementary Materials

The following supporting information can be downloaded at: https://www.mdpi.com/article/10.3390/pathogens13090818/s1, Figure S1: Viral RNA copies in serum samples of 70 patients plotted according to the infecting serotypes (A) and to types of infection with respect to each infecting serotype (B); Table S1: Primer Lists of real time RT-PCR for four serotypes of DENV, JEV and ZIKV; Table S2: The characteristics of dengue NS1 negative patients.
