# Supplementary material for: Performance of Fujifilm Dengue NS1 Antigen Rapid Diagnosis Kit Compared to Quantitative Real-Time Polymerase Chain Reaction
Source: Pathogens. 2024 Sep 23;13(9):818. doi: 10.3390/pathogens13090818 (PMC11434953; doi:10.3390/pathogens13090818)

Supplementary Figure 1.Viral RNA copies in serum samples of 70 patients plotted according to the infecting serotypes (A) and to types of infection with respect to each infecting serotype (B).

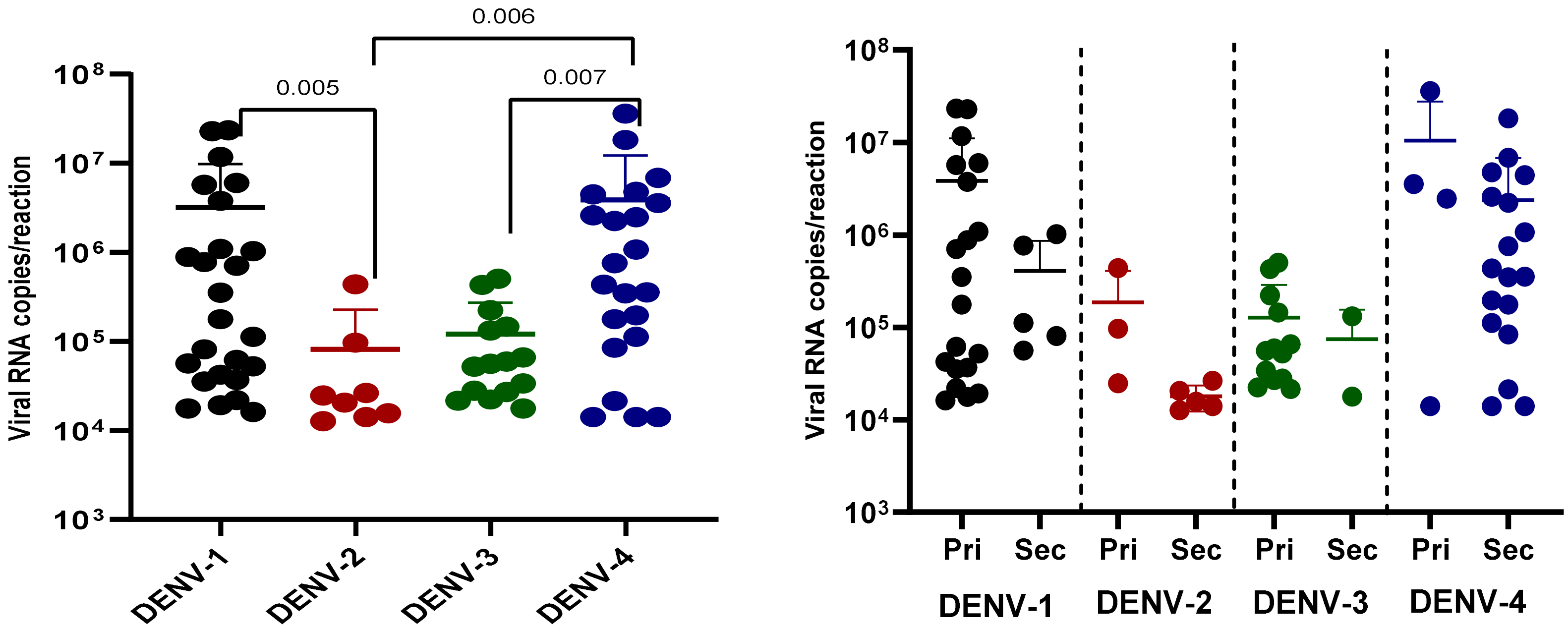

Supplement: Supplementary file 1 [file pathogens-13-00818-s001.zip › Supplementary Figure S1_pathogens.pdf]
