# Supplementary material for: Performance of Fujifilm Dengue NS1 Antigen Rapid Diagnosis Kit Compared to Quantitative Real-Time Polymerase Chain Reaction
Source: Pathogens. 2024 Sep 23;13(9):818. doi: 10.3390/pathogens13090818 (PMC11434953; doi:10.3390/pathogens13090818)
Supplement: Supplementary file 1 [file pathogens-13-00818-s001.zip › Supplementary Table S1_Pathogens.pdf]

Supplementary Table S1. Primer Lists of real time RT-PCR for four serotypes of DENV, JEV and ZIKV.

Virus	Gene Name	Sense	Sequences (5' - 3')	References
DENV-1	E	Forward	GAACATGGRACAAYTGCAACYAT	Ito et al, 2004
		Reverse	CCGTAGTCDGTCAGCTGTATTTCA	
		Probe	FAM-ACACCTCAAGCTCC-MGB	
DENV-2	E	Forward	ACACCACAGAGTTCCATCACAGA	Ito et al, 2004
		Reverse	CATCTCATTGAAGTCNAGGCC	
		Probe	FAM-CGATGGARTGCTCTC-MGB	
DENV-3	E	Forward	ATGAGATGYGTGGGAGTRGGAAAC	Ito et al, 2004
		Reverse	CACCACDTCAACCCACGTAGCT	
		Probe	FAM-AGATTTTGTGGAAGGYCT-MGB	
DENV-4	E	Forward	GGTGACRTTYAARGTHCCTCAT	Ito et al, 2004
		Reverse	WGARTGCATRGCTCCYTCCTG	
		Probe	FAM-CCAAGAGACAGGATGTGACAGTGCTRGGATC-TAMRA	
JEV	E	Forward	GGGCCTTCTGGTGATGTTT	Zhang et al, 2022
		Reverse	AAACCGCAGGAATVGTCAAT	
		Probe	FAM-TCGCAAGAGGTGGACGGCCA-MGB	Lanciotti et al, 2008
ZIKV	NS1	Forward	CCGCTGCCCAACACAAG	
		Reverse	CCACTAACGTTCTTTTGCAGACAT	
		Probe	FAM-AGCCTACCTTGACAAGCAGTCAGACACTCAA-MGB	
