# Supplementary material for: Performance of Fujifilm Dengue NS1 Antigen Rapid Diagnosis Kit Compared to Quantitative Real-Time Polymerase Chain Reaction
Source: Pathogens. 2024 Sep 23;13(9):818. doi: 10.3390/pathogens13090818 (PMC11434953; doi:10.3390/pathogens13090818)
Supplement: Supplementary file 1 [file pathogens-13-00818-s001.zip › Supplementary Table S2_Pathogens.pdf]

**Supplementary Table S2. The characteristics of dengue NS1 negative patients.**

| Sample ID | Age | Sex | Dengue Serotype | Days of fever | FF NS1 Kit | SD NS1 Kit |
|---|---|---|---|---|---|---|
| 1 | M | 6.5 | N/A | 6 | neg | neg |
| 2 | F | 14 | N/A | 5 | neg | neg |
| 3 | M | 11 | N/A | 2 | neg | neg |
| 4 | M | 9 | N/A | 5 | neg | neg |
| 5 | M | 6 | N/A | 5 | neg | neg |
| 6 | M | 5.5 | N/A | 6 | neg | neg |
| 7 | M | 5 | N/A | 8 | neg | neg |
| 8 | F | 6 | N/A | 7 | neg | neg |
| 9 | M | 7 | N/A | 4 | neg | neg |
| 10 | M | 10 | N/A | 7 | neg | neg |
| 11 | F | 3.8 | N/A | 5 | neg | neg |
| 12 | F | 8 | N/A | 5 | neg | neg |
| 13 | M | 9 | N/A | 4 | neg | neg |
| 14 | F | 9 | N/A | 6 | neg | neg |
| 15 | M | 3 | N/A | 6 | neg | neg |
| 16 | M | 10 | N/A | 4 | neg | neg |
| 17 | M | 9.3 | N/A | 5 | neg | neg |
| 18 | F | 8 | N/A | 5 | neg | neg |
| 19 | M | 2 | N/A | 4 | neg | neg |
| 20 | F | 2.5 | N/A | 4 | neg | neg |
| 21 | M | 6 | N/A | 4 | neg | neg |
| 22 | F | 10 | N/A | 6 | neg | neg |
| 23 | F | 8 | N/A | 3 | neg | neg |
| 24 | F | 7 | N/A | 3 | neg | neg |
| 25 | F | 12 | N/A | 6 | neg | neg |
| 26 | F | 3.5 | N/A | 3 | neg | neg |
| 27 | F | 10 | N/A | 5 | neg | neg |
| 28 | F | 4 | N/A | 3 | neg | neg |
| 29 | F | 4.5 | N/A | 5 | neg | neg |
| 30 | F | 11 | N/A | 4 | neg | neg |
| 31 | M | 6.6 | N/A | 5 | neg | neg |
| 32 | F | 6 | N/A | 4 | neg | neg |
| 33 | M | 5.5 | N/A | 5 | neg | neg |
| 34 | M | 7 | N/A | 4 | neg | neg |
| 35 | F | 7 | N/A | 5 | neg | neg |

| | | | | | | |
|---|---|---|---|---|---|---|
| 36 | M | 5 | N/A | 5 | neg | neg |
| 37 | M | 0.8 | N/A | 5 | neg | neg |
| 38 | M | 7 | N/A | 5 | neg | neg |
| 39 | M | 3.8 | N/A | 5 | neg | neg |
| 40 | F | 3 | N/A | 4 | neg | neg |
| 41 | M | 6 | N/A | 6 | neg | neg |
| 42 | M | 6 | N/A | 4 | neg | neg |
| 43 | F | 12 | N/A | 5 | neg | neg |
| 44 | F | 4 | N/A | 6 | neg | neg |
| 45 | F | 11 | N/A | 6 | neg | neg |
| 46 | F | 5 | N/A | 4 | neg | neg |
| 47 | M | 9 | N/A | 5 | neg | neg |
| 48 | F | 7.5 | N/A | 5 | neg | neg |
| 49 | M | 9 | N/A | 7 | neg | neg |
| 50 | F | 7 | N/A | 6 | neg | neg |
| 51 | F | 9 | N/A | 3 | neg | neg |
| 52 | F | 7 | N/A | 1 | neg | neg |
| 53 | F | 10 | N/A | 4 | neg | neg |
| 54 | M | 12 | N/A | 3 | neg | neg |
| 55 | M | 5.3 | N/A | 4 | neg | neg |
| 56 | F | 5 | N/A | 5 | neg | neg |
| 57 | M | 5 | N/A | 2 | neg | neg |
| 58 | M | 3.5 | N/A | 5 | neg | neg |
| 59 | F | 12 | N/A | 5 | neg | neg |
| 60 | F | 6 | N/A | 4 | neg | neg |
| 61 | F | 7 | N/A | 6 | neg | neg |
| 62 | M | 11 | N/A | 6 | neg | neg |
| 63 | F | 4 | N/A | 6 | neg | neg |
| 64 | M | 10 | N/A | 7 | neg | neg |
| 65 | F | 6 | N/A | 6 | neg | neg |
| 66 | M | 8.5 | N/A | 4 | neg | neg |
| 67 | F | 7 | N/A | 4 | neg | neg |
| 68 | M | 5 | N/A | 5 | neg | neg |
| 69 | M | 7 | N/A | 5 | neg | neg |
| 70 | M | 7 | N/A | 2 | neg | neg |

N/A= not detected.

neg= NS1 negative.
